# Progression of Antiviral Agents Targeting Viral Polymerases

**DOI:** 10.3390/molecules27217370

**Published:** 2022-10-29

**Authors:** Siqi Peng, Huizhen Wang, Zhengtao Wang, Qingzhong Wang

**Affiliations:** Institute of Chinese Materia Medica, Shanghai University of Traditional Chinese Medicine, Shanghai 201203, China

**Keywords:** viral DNA and RNA polymerase, antiviral drugs, inhibitors, natural products

## Abstract

Viral DNA and RNA polymerases are two kinds of very important enzymes that synthesize the genetic materials of the virus itself, and they have become extremely favorable targets for the development of antiviral drugs because of their relatively conserved characteristics. There are many similarities in the structure and function of different viral polymerases, so inhibitors designed for a certain viral polymerase have acted as effective universal inhibitors on other types of viruses. The present review describes the development of classical antiviral drugs targeting polymerases, summarizes a variety of viral polymerase inhibitors from the perspective of chemically synthesized drugs and natural product drugs, describes novel approaches, and proposes promising development strategies for antiviral drugs.

## 1. Introduction

Viruses have a relatively simple life structure which causes them to be hard to eliminate but also quick to mutate, and infect hosts again and again [1]. There are many viral infection diseases, and more than 70% of infectious diseases affecting humans are caused by viruses, including the seasonal influenza virus, human immunodeficiency virus (HIV)-related AIDS, chronic hepatitis B virus (HBV), and sudden viral diseases like Ebola, Middle East respiratory syndrome coronavirus (MERS-CoV), severe acute respiratory syndrome (SARS), highly pathogenic avian influenza, and others [2,3]. Antiviral drug studies are more challenging and have experienced slowing progress due to the parasitic properties of viruses, which require host cells [4]. Antiviral drug development started in the 1960s, more than 30 years after the creation of antibacterial medications. Iodine, the first antiviral medicine, received approval in June 1963, ushering in a new era in antiviral drug research [3,5]. More than 90 antiviral medications, including over 20 anti-Hepatitis C virus (HCV) medications and 40 anti-HIV medications, have received official marketing approval between 1963 and 2016 [3]. In particular, the introduction of sofosbuvir as an HCV NS5B polymerase inhibitor marked a significant turning point in the treatment of HCV in 2013, which encouraged and accelerated global research on antiviral medicines. In the past 30 years, significant advancements have been achieved in the study and creation of novel antiviral medications, with an emphasis on antiretroviral and anti-HCV medications [6]. Most current antiviral medications are direct antiviral medications, which are designed to specifically target viral enzymes. These medications have the benefits of a clear target, high levels of specificity, and potent action, but the drawbacks of a limited antiviral spectrum and drug resistance [7]. The viral life cycle mainly includes these processes: adsorption and penetration, uncoating, early transcription, the early stage of translation, viral genome replication, late transcription, late translation, viral assembly, release, etc., and theoretically all stages of the viral life cycle could be potential targets for antiviral drugs [8]. Based on viral DNA/RNA polymerase, we summarized not only the antiviral drugs that have been marketed, but also the synthesized drugs and natural products under development, especially the potential compounds that have been recently widely reported (Table 1). In the meantime, we described novel discovery methods and strategies, such as nucleic acid degradation, protein degradation, RNA interference application drugs, and capsid protein assembly regulators.

## 2. Common Viruses and Key DNA/RNA Polymerases

According to the Baltimore Classification, viruses can be divided into seven groups based on the source of the viral mRNA: (1) Double-stranded DNA viruses (dsDNA) such as herpesviridae, cytomegalovirus, adenoviruses, and poxvirus; (2) Single-stranded DNA viruses (ssDNA) such as parvoviruses; (3) Double-stranded RNA viruses (dsRNA) such as reoviruses; (4) Positive-strand RNA viruses (+ssRNA) such as coronavirus, the Hepatitis C virus, Flavivirus, and Norovirus; (5) Negative-strand RNA viruses (−ssRNA) such as the Influenza virus, filovirus, respiratory syncytial viruses, and rhabdoviruses; (6) Single-stranded RNA retroviruses (ssRNA RT) such as human immunodeficiency virus, leukemia virus, and sarcoma virus; and (7) Double-stranded RNA retroviruses (dsRNA RT) such as hepadnaviridae and Caulimoviridae. We focus on the viruses which have mature antiviral drugs targeting DNA/RNA polymerase. Therefore, the selection criteria focus on those viruses for which there are marketed antiviral drugs that target the viral synthase. Thus, we searched the literature for records which have been reported up until August 2022; the keywords mainly included “Viral DNA/RNA polymerase”, “Antiviral drugs”, and “Antiviral natural products”. We excluded drugs that were terminated, non-progressive, and withdrawn, and selected antiviral drugs that target the DNA/RNA synthases of four common RNA viruses that have been approved for marketing by the FDA, EMA, NMPA, PMDA, etc., or had clinical trial results in CTGOV, CTR, WHO, and were reported in the literature in PubMed.

### 2.1. HBV

The tiny DNA virus known as HBV primarily causes viral hepatitis B disease by infecting host hepatocytes [9]. HBV replicates through reverse transcription of an RNA intermediate and its genetic material consists of a 3.2-kb relaxed partial double-stranded circular DNA (rcDNA) [10]. In the host cells, DNA polymerase converts it into covalently closed circular DNA (cccDNA), which acts as a template for viral transcription and promotes viral multiplication [11]. The HBV polymerase is a specialized reverse transcriptase with multiple active functions that is also essential for HBV to infect host cells. HBV polymerase has four functional domains: the terminal protein domain (TP), the spacer domain (SD), the reverse transcriptase domain (HBV RT), and the ribonuclease H domain (HBV RNase H) [12]. HBV RT is the major functional domain of HBV polymerase, which has reverse transcriptase and DNA polymerase activity and can reverse transcribe HBV RNA into negative-stranded DNA, and then use this as a template for the synthesis of positive-stranded DNA [13]. During viral replication, HBV RNase H is responsible for degrading pregenomic RNA (pgRNA), which promotes the synthesis of positive-stranded DNA [14].

Since cccDNA is stable, as of now HBV infection can only be controlled and it cannot be cured [15]. The primary cause of severe liver damage, including liver cirrhosis and hepatocellular carcinoma, is chronic HBV infection [16]. At present, three types of anti-HBV medications have been approved: (1) Telbivudine and entecavir as DNA polymerase inhibitors, which act on DNA polymerase to prevent the replication of viral genetic material and viral amplification [17]; (2) the reverse transcriptase inhibitor lamivudine, a type of nucleoside reverse transcriptase inhibitor (NRTI), which can produce an antiviral effect by blocking the HBV polymerase [18], as well as tenofovir and adefovir, which work as NRTI agents by blocking reverse transcriptase (RT) and cannot reproduce in the body [19]; and (3) PEGylated interferons, such as PEGIFNa-2a and PEGIFNa-2b, which stimulate immune cells (CD8 cells and natural killer T cells), improve non-cytolytic viral clearance by cytokines or cytolysis of infected cells, and enhance the expression of innate antiviral genes and proteins [20].

### 2.2. HIV

HIV, as a retrovirus in the lentivirus family, can cause acquired immunodeficiency syndrome and T-cell immunological insufficiency resulting in infection (AIDS) [21]. HIV RT and HIV integrase are two crucial antiviral targets that act during the viral biosynthesis stage [22]. HIV RT is an RNA-dependent DNA polymerase that can produce complementary DNA from RNA templates, which is a crucial step in the HIV life cycle known as reverse transcription [23]. The p66 subunit domain of HIV RT contains three catalytic carboxylates (D110, D185, D186), which are the key active sites for the catalytic function of the polymerase and are also used as key sites for the development of NRTIs [24,25,26,27]. A hydrophobic pocket about 10 to 15 Å below the polymerase active site is the allosteric binding site for non-nucleoside reverse transcriptase inhibitors (NNRTIs) [28].

Another key target, HIV integrase, is responsible for the integration of the full-length linear HIV DNA genome into host chromatin, as well as for 3’-end processing and catalytic activity for strand transfer [29]. The catalytic site of HIV integrase and the conserved catalytic triad Asp64–Asp116–Glu152 are often used as key sites for the development of targeted integrase strand transfer inhibitors [30]. 

### 2.3. HCV

HCV is a positive-sense, single-stranded, enveloped RNA virus of the flaviviridae family that can lead to acute hepatitis or chronic disease [31]. HCV has a 9.6-kb genome, and its encoded proteins are cleaved by host and viral proteases into 10 mature viral proteins with different functions [32]. HCV NS5B polymerase is an RNA-dependent RNA polymerase (RdRp) primarily responsible for HCV RNA synthesis and genome replication [33]. In the palm domain of RNA polymerase, the catalytic site of NS5B uses HCV positive-strand RNA as a template for the polymerization of ribonucleoside triphosphates, which can be blocked by competitive binding of nucleotide inhibitors such as sofosbuvir to interrupt HCV RNA synthesis [34,35]. Although multiple NS5B inhibitors have been reported, it is worth noting that dasabuvir (ABT-333) remains the only FDA-approved non-nucleoside NS5B inhibitor [36].

### 2.4. Influenza Virus

The influenza virus is an enveloped single-stranded negative-sense RNA virus of the family orthomyxoviridae that causes seasonal influenza and is spread by droplets [37,38]. It causes an infectious respiratory disease with symptoms such as a high fever, headache, arthralgia, and muscle discomfort [39]. Influenza A virus has 18 different hemagglutinin subtypes and 11 different neuraminidase subtypes. Influenza polymerase can hijack the host mRNA transcription system to synthesize viral RNA. The endonuclease of the two subunits of influenza polymerase PA and the catalytic site of PB1 are often used to develop related anti-influenza virus drugs.

### 2.5. Severe Acute Respiratory Syndrome Coronavirus 2 (SARS-CoV-2)

Coronaviruses are a type of single-stranded positive-sense RNA viruses with envelopes in the family coronaviridae, which are transmitted in the air as droplets [40]. Seven types of coronaviruses have been found to infect humans [41]. In recent years, COVID-19, a novel coronavirus caused by SARS-CoV-2, has caused a global pandemic, which causes severe lower respiratory disease and ultimately death [42]. Like influenza viruses, the replication of coronavirus genetic material is mediated by the RdRp complex [43]. The core catalytic site of the coronavirus RNA polymerase is NSP12, which binds to NSP7 and NSP8 to stabilize its own closed conformation [44,45]. Meanwhile, nucleoside inhibitors (NI) can bind RdRp competitively with natural nucleoside substrates, which can inactivate the RdRp enzyme and cause abnormal termination of RNA synthesis [46].

## 3. Viral DNA/RNA Synthases Inhibitors

After the virus enters the host cell, the host cell is used to synthesize the virus’ own nucleic acid and protein [47]. Most of the antiviral drugs that are clinically used act on the viral replication stage [48].

### 3.1. Influenza Virus RNA Polymerase Inhibitor

The replication for most viral RdRp is processed within the host cells. Influenza virus RdRp consists of three subunits encoded by the virus: PB1, PB2, and PA [49]. The naked viral genomic RNA must be combined with nucleic proteins to form a complex that serves as a template to initiate viral genome replication and transcription by RdRp. Studies have shown that the three subunits of influenza virus RdRp can enter the nucleus individually [50,51,52]. First, RdRp is transported by the nuclear localization signal (NLS) of the nucleoprotein (NP) into the host’s nucleus for assembly [53]. After assembly, the replication process of the influenza virus’ genetic material begins. Since the influenza virus cannot produce 5’-cap primers on its own, its PB2 subunit captures the 5’-cap structure of host cell RNA through a “cap capture” mechanism [54]. After replication is completed, the product is exported through a separate channel for viral mRNA synthesis [55]. Different types of inhibitors targeting the PB1, PB2, and PA subunits are discussed in detail below [56,57].

#### 3.1.1. PA Inhibitors

At present, the PA inhibitor baloxavir marboxil, jointly developed by Shionogi and Roche, is used for the treatment of influenza A and B in individuals over the age of 12 [58]. Since there is no similar mechanism and corresponding protease in host cells, baloxavir acts as a novel CAP-dependent nucleic acid endonuclease inhibitor, and can selectively block the transcription process of the influenza virus without affecting host cells [59,60]. The results of clinical trials show that baloxavir can significantly improve the time of symptom relief and that this drug is well-tolerated [61].

#### 3.1.2. PB1 Inhibitors

Among the PB1 inhibitors, ribavirin and favipiravir have entered the clinical research stage due to their good antiviral activity [62]. Although both compounds contain bases in their structures, their antiviral mechanisms are not the same [63]. Ribavirin is a broad-spectrum antiviral drug widely accepted as a competitive inhibitor of host monophosphate dehydrogenase (IMPDH) [64]. IMPDH can catalyze the conversion of guanosine monophosphate (GMP) to guanine triphosphate (GTP), and inhibition of this enzyme will reduce the content of GTP in cells, resulting in an imbalance in nucleotide concentration, thereby inhibiting viral protein synthesis and exerting an antiviral effect [65]. Favipiravir belongs to a class of purine analogs that need to be rapidly converted into triphosphate form in vivo, and it exerts an antiviral effect by simulating competitive inhibition of RNA polymerase activity by GTP [66,67]. At the same time, it can also be incorporated into viral genes and exert antiviral effects by inducing lethal mutations [68]. In addition to being anti-influenza virus, favipiravir is effective against a variety of RNA viruses such as Lassa fever virus, Rift Valley fever virus, Hantavirus, Flaviviridae, West Nile virus, Zika virus, Chikungunya virus, Ebola virus, etc [66].

#### 3.1.3. PB2 Inhibitors

Pimodivir, also known as VX-787, is a typical representative of the PB2 cap-binding domain inhibitors [69]. Based on phase III clinical study data, pimodivir did not show better efficacy than the existing standard compound, so Janssen decided to stop its clinical development as an anti-influenza A virus drug [70].

#### 3.1.4. Protein–Protein Interaction Inhibitors

**Figure 1:**
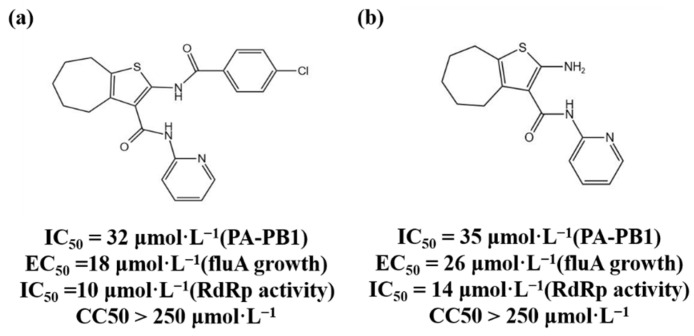
(**a**,**b**) Chemical structures of Protein–protein interaction inhibitors and their antiviral activities.

The three subunits of RdRp are non-covalently combined into a functional complex, so blocking the interaction between the subunits can effectively inhibit the activity of RdRp [71]. The polymerase inhibitors developed based on this mechanism are called protein–protein interaction inhibitors (PPI inhibitors) [72]. At present, the most commonly studied PPI inhibitors are PA-PB1 inhibitors. Massari et al. (2013) found that cycloheptathiophene-3-carboxamide compounds had weak PA-PB1 inhibitory activity but no antiviral activity through ELISA experiments, and further modified cycloheptathiophene-3-carboxamide compounds by considering their structure–activity relationship. They also synthesized 35 compounds, of which 1 and 2 had the strongest activity and whose structures are shown in Figure 1; their IC_50_ values were 32 µmol/L and 35 µmol/L, respectively, and they had no cytotoxicity at the concentration of 250 µmol/L. These two compounds became potential PPI inhibitors [73].

### 3.2. HCV-related Inhibitors

The NS5B polymerase encoded by the non-structural gene NS5B is an enzyme of RdRp [74]. Its structure has the “right-hand” conformation of a typical RNA polymerase. The “palm subdomain“ is the central part of the polymerase and is the catalytic center of genome replication; the “fingers subdomain“ is responsible for capturing the nucleotide triphosphates required for replication; and the “thumb subdomain“ coordinates the initiation and elongation of RNA replication [75]. HCV replicates by using the viral genome’s single-stranded RNA as a template and joining the replicon created by the NS5B polymerase [76]. Because human cells lack similar enzymes, NS5B polymerase is a promising antiviral drug target. HCV NS5B polymerase inhibitors are divided into two categories: NIs and non-nucleoside inhibitors (NNIs) [77].

#### 3.2.1. NIs

Sofosbuvir is a uridine analog prodrug, which can specifically inhibit HCV NS5B polymerase activity and is used for antiviral therapy in patients with chronic hepatitis C. The mechanism of action involves sofosbuvir being converted into the active uridine triphosphate under the action of intracellular phosphokinase, which competes with intracellular nucleotide phosphate as a substrate for NS5B polymerase and is incorporated into newly synthesized RNA chain, terminating the elongation of the HCV RNA chain, thereby inhibiting the replication of HCV [78]. Sofosbuvir has no inhibitory activity on human DNA and RNA polymerase, nor on mitochondrial RNA polymerase, so it has strong specificity [79]. Meanwhile, Sofosbuvir is a “pan-genotype” anti-HCV drug which not only inhibits HCV genotype 1 disease but is also effective against other genotypes of HCV infection.

#### 3.2.2. NNIs

NNIs bind to the allosteric site of NS5B polymerase, resulting in a change in enzyme conformation, thereby inhibiting the activity of NS5B polymerase and exerting an antiviral effect [80,81]. Compared with NIs, NNIs have a lower genetic barrier and are prone to drug resistance and relapse after drug withdrawal, and cannot have antiviral effects on all genotypes [82]. Among them, dasabuvir sodium hydrate was approved by the European Medicines Agency (EMA) in January 2015 as an NNI-type drug.

#### 3.2.3. NS5A Inhibitors

The HCV nonstructural protein NS5A is one of the components of the viral RNA replication complex [83]. It has not yet been found to have enzymatic activity, but it is essential for HCV RNA replication and is also related to the INF response [84]. NS5A inhibitors may exert anti-HCV effects by inhibiting the hyperphosphorylation of NS5A or altering the subcellular localization of NS5A [85]. Daclatasvir is a representative NS5A inhibitors which has a strong antiviral effect and is a pan-genotype drug [86].

### 3.3. HIV-1 Reverse Transcriptase Inhibitors

After retroviruses such as HIV-1 enter cells, they first synthesize RNA-DNA complex strands under the action of reverse transcriptase, and then the RNA strands are hydrolyzed to synthesize double-stranded DNA [87]. Under the action of the enzyme, this DNA is embedded into the genome of the host cell and replicated with the transcription and translation machinery of the host cell [88]. HIV-1 RT is the product of HIV-1 pol gene and consists of p51 and p66 subunits; the RT active center is located in the p66 subunit, which has RNA-dependent DNA polymerase activity [87,89]. Its DNA polymerase domain also has a “right-hand” conformation, similar to the RNA virus polymerase. It has four subdomains: the finger, thumb, palm, and linker domains. It is mainly responsible for the synthesis of double-stranded DNA using HIV single-stranded RNA as a template. The finger, thumb, and palm subdomains together constitute the active site, which occurs so that the enzyme is able to locate the template-primer accurately, and the palm domain contains the key site of DNA chain replication and elongation [90]. There are currently two types of reverse transcriptase inhibitors: NRTIs and NNRTIs [91].

#### 3.3.1. NRTIs

NRTIs are analogs of deoxynucleotides, the DNA RT substrates for the synthesis of HIV-1. In vivo, NRTIs are converted into active nucleoside triphosphate derivatives, which compete with the natural deoxynucleoside triphosphate to bind HIV-1 RT to inhibit RT activity or terminate the RNA chain [92,93]. Currently approved NRTIs include zidovudine (AZT), didanosine (ddI), zalcitabine (ddC), stanvudine (d4T), lamivudine (3TC), abacavir (ABC), emtricitabine [(-)FTC], tenofovir (TFV), etc. [94].

#### 3.3.2. NNRTIs

NNRTIs can directly combine with the p66 hydrophobic region of HIV-1 RT to change the conformation of the enzyme protein and inhibit the enzyme’s activity [87,95]. Unlike NRTIs, NNRTIs are not directly incorporated into DNA strands and do not require phosphorylation, and can function in quiescent and activated cells [96]. NNRTIs currently approved for clinical use include nevirapine (NVP), delavirdine (DLV), efavirenz (EFV), etravirine (TMC-125), and rilpivirine (RPV) [97].

#### 3.3.3. HIV-1 Integrase Inhibitors

HIV-1 integrase catalyzes the integration of the HIV-1 virus reverse transcription product cDNA into the human host genome [98]. HIV-1 integrase is encoded by the 3’-end of the viral pol gene and contains 288 amino acid residues [99]. The integrase has 3’ cleavage endonuclease activity or strand transfer activity in vivo [100]. Inhibition of the HIV-1 integration process can be achieved by inhibiting the endonuclease activity or strand transfer activity of the integrase. There are currently four HIV-1 integrase inhibitors approved for clinical mediation, namely raltegravir, elvitegravir, dolutegravir, and bictegravir, and all four drugs act on the chain transfer process [101].

#### 3.3.4. SARS-CoV-2 Enzyme Inhibitors

The structure of the polymerase complex of SARS-CoV-2 includes one NSP12, one NSP7, and two NSP8 subunits, which are required to complete the replication process of the coronavirus RNA [102]. The core catalytic site of the coronavirus polymerase is NSP12, and its RdRp domain is present in the standard “right-hand” conformation, including the three finger, thumb, and palm subdomains [103]. The RdRp associates with additional non-structural proteins to form a replication-transcription complex that carries out RNA synthesis, capping, and proofreading [104]. Remdesivir was originally a drug used for the treatment of hepatitis C and was later shown to be a broad-spectrum antiviral drug with a delayed chain termination mechanism of action [105,106]. Remdesivir is also being evaluated as an anti-coronavirus drug, and is currently the only drug approved by the US FDA for the treatment of patients with COVID-19. Favipiravir was originally used to treat RNA virus infections such as Ebola and influenza, but a randomized clinical trial found that the drug can bind to the RdRp metal catalytic site of SARS-CoV-2 and produce inhibitory activity [107]. Therefore, favipiravir has been urgently approved for the treatment of mild COVID-19 in several countries [108]. At present, the drug has entered phase 3 clinical trials for the treatment of COVID-19 in many countries [109]. Recent studies have found that suramin, an NNI, can effectively inhibit the activity of SARS-CoV-2 RdRp and prevent the virus from entering cells [110]. It has been proved that the anti-COVID-19 mechanism involves two symmetrical suramin molecules binding to RdRp and preventing RNA templates and primers from binding [111]. It binds to the active site and prevents nucleotide triphosphates from entering the catalytic site, thereby inhibiting the growth of SARS-CoV-2 [112].

## 4. Research and Development Strategies for Novel Antiviral Drugs

Due to the increasing cost of drug research and development, traditional drug random screening strategies and blind optimization of lead compounds consume a lot of resources and time. In recent years, some new research strategies have been developed, which are listed as follows:

### 4.1. Nucleic Acid Degradation

Ribonuclease targeting chimera (RIBOTAC) technology converts RNA-binding molecules into RNA-degrading molecules to degrade the viral genome by combining RNA-binding molecules with a small molecule and activating ribonuclease L (RNase L) [113]. Haniff et al. (2020) verified through a series of experiments that compound C5 can stabilize the frameshift element and significantly inhibit the frameshift ability of the SARS-CoV-2 frameshift element. At the same time, the structure of compound 39 was modified with the help of RIBOTAC technology, that is, compound 39 was connected to a small molecule that can recruit RNase L through a linking chain of suitable length to achieve the purpose of directional degradation of SARS-CoV-2 mRNA [114].

### 4.2. Protein Degradation

Targeting protein degradation chimera (proteolysis-targeting chimera, PROTAC) molecules can target degradation of proteins [115]. PROTAC bifunctional small molecules can bring target proteins and E3 ubiquitin ligases closer together, induce ubiquitination of target proteins and subsequent degradation through the ubiquitin-proteasome pathway [116]. Yang et al. (2022) used PROTAC technology to develop anti-HCV molecules, which can induce viral proteasome degradation [117]. The PROTAC molecule combined with the ligand of CRL4CRBN can induce HCV NS3/4A protease degradation, proving that protein degradation contributes to its antiviral activity [118].

### 4.3. RNA Interference Application Drugs

RNA interference (RNAi) refers to the specific gene expression silencing mediated by double-stranded RNA [119]. ARC-520 is an RNA interference (RNAi)-based drug for the treatment of chronic hepatitis B [120]. It can act on HBV covalently closed circular DNA (cccDNA) transcription to degrade mRNA [121]. Nonclinical toxicology studies in primates found that ARC-520 may be potentially toxic, and temporarily halted clinical trials of this drug [122]. The HBV RNAi drugs currently under development include ARB-1467 (Phase II clinical trial), RG-6004 (Phase I clinical trial), GSK-3389404, and GSK-3228836 (Phase II clinical trial) [123,124,125].

### 4.4. Capsid Protein Assembly Regulators

The HBV capsid protein assembly regulator can inhibit the replication of the HBV virus by destroying the function of the capsid [126]. The HBV capsid can not only protect the viral genome encapsulated in the capsid, but also promote the reverse transcription of pgRNA to form DNA [127,128]. Heteroaryldihydropyrimidine (HAP) compounds used as HBV capsid assembly modulators (CpAM) can enhance the hydrophobic interaction between adjacent core protein dimers, alter the dimerization angle between the two substances, change their assembly kinetics, and accelerate the degradation of capsid proteins [129,130]. Electron microscopy results show that HAP compounds can promote the formation of large and irregular particles around which misassembled capsids cannot properly wrap pgRNA, and prevent HBV replication [131]. Representative HBV capsid protein assembly regulators include Bay41-4109 (first generation), GIS-4 (second generation, phase 1 clinical trial), and HAP-R10 (third generation, phase I clinical trial) [132,133].

## 5. Natural Products as Antiviral Drugs

Studies have found that many natural products can inhibit the activities of integrase, RT, and protease to achieve antiviral activity; these natural products include flavonoids, polyphenols, alkaloids, coumarins, terpenes, peptides, etc. Most of these antiviral natural products are flavonoids and polyphenols, followed by terpenoids (mainly diterpenes and triterpenes), and there are fewer alkaloid inhibitors [134]. Several natural products have been identified as potent inhibitors of the enzymatic activity of HIV enzymes, including RT and integrase [135], such as: Kuwanon-L isolated from the black mulberry tree Morus nigra L. (Morus nigra); Patentiflorin A, isolated from Justicia gendarussa Burm.f. (Justica gendarussa); and many plant-based compounds extracted from Calophyllum lanigerum var. austrocoriaceum (Calophyllum lanigerum), which have anti-RT and anti-integrase activities equivalent to those of highly active antiretroviral drugs [136,137,138]. The aqueous extract of E. alba and its active isolates inhibit in vitro HCV NS5B activity and HCV RdRp activity [139]. 3-hydroxycaruilignan C (3-HCL-C) isolated from Wisteria sinensis can also induce IFN-stimulated response element transcription and IFN-dependent antiviral gene expression to interfere with HCV replication [140]. Recently, we integrated computer-aided drug development (CADD), deep learning (DL), and similarity-based clustering methods and experimental validation to perform high-throughput screening of large-scale molecular databases and obtain potential anti-SARS-CoV-2 virus natural products. Five different natural products, including narcissoside, kaempferol-3-O-gentiobioside, rutin, vicin-2, and isoschaftoside, can target 3clpro, with the potential to inhibit SARS-CoV-2 activity [141].

## 6. Discussion

This present review mainly focused on the recent progress and development of classical antiviral drugs targeting polymerases, supplemented with a variety of viral polymerase inhibitors from the perspective of chemically synthesized compounds and natural products, and also introduced novel approaches based on characteristic of viral polymerase. In recent years, the treatment of chronic viral diseases such as HIV and HBV has been greatly improved by the intervention of antiviral drugs. Unfortunately, a complete cure of these viral diseases has not yet been achieved, and there is still the possibility of recurrence or outbreak, and long-term medication is required. In addition, drug resistance is the biggest challenge faced in antiviral therapy [142,143]. Different antiviral drugs have different mechanisms of drug resistance. In addition, when a new epidemic suddenly appeared, there were still no available effective drugs. Faced with such complex challenges, we proposed the following development strategies for antiviral drugs: (1) Drug Repurposing: Repurposed drugs are now often used to treat new viruses with approved drugs, or those approved drugs are structurally modified. For example, remdesivir was developed as an anti-HCV drug and was recently found to have activity against SARS-COV-2 [144]. Favipiravir was originally used to treat RNA virus infections such as Ebola and influenza, but a randomized clinical trial found that favipiravir can bind to the RdRp catalytic site of SARS-CoV-2 to produce active inhibition [145]. In addition, different viruses may share common therapeutic targets because of the similar structure of many enzymes involved in infection among different viruses. For example, all the polymerases of RNA viruses have a typical “right-handed” conformation [112]. For the effective and approved RNA polymerase inhibitors, the functional group of the compound can be modified by considering the structure–activity relationship, or the structure can be replaced to make the drug more active [146]. (2) Prodrug strategy: Prodrug strategy-based nucleoside drugs play an important role in antiviral therapy. However, many nucleoside drugs have defects such as low oral bioavailability, rapid metabolism, and high toxicity [147]. The use of prodrug strategies can not only optimize the pharmacological activity of these drugs, but also represent an important direction in the development of nucleoside antiviral drugs. For example, valacyclovir and valganciclovir are valine ester prodrugs of acyclovir and ganciclovir, respectively [148,149]. Antiviral drugs such as tasvir, sofosbuvir, adefovir, tenofovir, and abacavir, have adopted the prodrug strategy [150]. (3) Natural products: Natural products have a wide range of sources and unique structures, which are important sources for the discovery of antiviral drug lead compounds and drug candidates [151]. Natural products have shown promising antiviral activity against many RNA viruses that cause endemic and pandemic infections [152,153]. A variety of active phytochemicals, including coumarins, flavonoids, terpenoids, organosulfur compounds, lignans, polyphenols, saponins, proteins and peptides, have been found to affect cellular function, membrane permeability, and viral replication. At the same time, natural antiviral drugs can provide broad-spectrum antiviral activity and reduce drug resistance [154]. At present, by using computer-aided virtual screening of natural products libraries, more natural products with effective antiviral activity are expected to be discovered [155,156]. With the deepening of basic research and the continuous innovation of chemical and clinical research, the pace of antiviral drug development has been greatly accelerated. In addition, the cure of HCV provides an important experience for the other chronic viral diseases [157]. In the future, more antiviral drugs will be successfully developed, and more viral diseases will be cured.

## Figures and Tables

**Table 1:** Summary of approved drugs and compounds in development targeting DNA/RNA enzymes of common viruses.

Viruses	Targets	Approved Drugs	Novel Inhibitors
Human immunodeficiency virus (HIV)	HIV-1 reverse transcriptase (NRTI)	Zidovudine	MK-8583
		Didanosine	Rovafovir etalafenamide
		Stavudine	Islatravir (mk-8591)
		Lamivudine	Tenofovir Alafenamide
		Emtricitabine	Tenofovir amibufenamide
		Abacavir Sulfate	Tenofovir Alafenamide Fumarate
		Apricitabine	Abacavir hydroxyacetate
		Tenofovir Disoproxil Fumarate	
		Tenofovir Succinate	
		Tenofovir	
		Azvudine	
	HIV-1 reverse transcriptase (NNRTI)	Nevirapine	VM-1500A
		Delavirdine mesylate	Dapivirine
		Efavirenz	IQP-0528
		Etravirine	IQP-0528
		Elsulfavirine	
		Ainuovirine	
		Doravirine	
		Rilpivirine Hydrochloride	
	HIV-1 integrase	Raltegravir Potassium	Bictegravir
		Cabotegravir sodium	Fipravirimat
		Dolutegravir Sodium	STP-0404
			GSK3732394
Hepatitis C virus (HCV)	NS5B polymerase (NI)	Sofosbuvir	Adafosbuvir
			Bemnifosbuvir
	NS5B polymerase (NNI)	Dasabuvir Sodium Hydrate	Radalbuvir
			GSK-2878175
			TMC-647055
			CC-31244
	NS5A inhibitors	Emitasvir Phosphate	Ledipasvir
		Coblopasvir hydrochloride	
		Elbasvir	
		Daclatasvir Dihydrochloride	
	Cyclophilin A (Cyp)		SCY-635
			Alisporivir
			EDP-494
Human influenza virus	RNA polymerase (PA)	baloxavir marboxil	
	RNA polymerase (PB2)		Onradivir
	RNA polymerase (PB1)	Favipiravir	
Severe acute respiratory syndrome coronavirus 2 (SARS-CoV-2)	RdRP polymerase (Nucleoside)	Remdesivir	Remindevir
		Favipiravir	Galidesivir
			GS-441524

## Data Availability

Not applicable.
